# Adverse biobehavioral effects in infants resulting from pregnant rhesus macaques’ exposure to wildfire smoke

**DOI:** 10.1038/s41467-022-29436-9

**Published:** 2022-04-01

**Authors:** John P. Capitanio, Laura A. Del Rosso, Nancy Gee, Bill L. Lasley

**Affiliations:** 1 California National Primate Research Center, University of California, Davis, CA USA; 2 Department of Psychology, University of California, Davis, CA USA; 3 Center for Health and the Environment, University of California, Davis, CA USA

**Keywords:** Psychology, Psychology and behaviour

## Abstract

As wildfires across the world increase in number, size, and intensity, exposure to wildfire smoke (WFS) is a growing health problem. To date, however, little is known for any species on what might be the behavioral or physiological consequences of prenatal exposure to WFS. Here we show that infant rhesus monkeys exposed to WFS in the first third of gestation (n = 52) from the Camp Fire (California, November, 2018) show greater inflammation, blunted cortisol, more passive behavior, and memory impairment compared to animals conceived after smoke had dissipated (n = 37). Parallel analyses, performed on a historical control cohort (n = 2490), did not support the alternative hypothesis that conception timing alone could explain the results. We conclude that WFS may have a teratogenic effect on the developing fetus and speculate on mechanisms by which WFS might affect neural development.

## Abstract (web summary)

Little is known about the consequences of prenatal exposure to wildfire smoke on biobehavioural outcomes. Here, the authors show that infant rhesus monkeys exposed early in gestation to wildfire smoke from the 2018 Camp Fire in California show more inflammation, blunted cortisol and altered behaviour outcomes compared to non-exposed animals.

## Introduction

As the climate changes and the forest under-story continues to accumulate, the number, size, and intensity of wildfires around the world have changed. Whereas wildfires in the western United States burned approximately 3.3 million acres per year in the 1990s, since the 2000s the amount of acreage burned per year has doubled^1^. In California, five of the six largest wildfires in its history were recorded in 2020^2^, and in November, 2018, California’s deadliest wildfire, the Camp Fire originating in Butte County, CA, occurred^3^. The Camp Fire began on 8 Nov 2018, and the smoke plume from the fire, which traveled hundreds of miles, resulted in elevated PM_2.5_ (particulate matter of 2.5 microns or less in aerodynamic diameter) levels for a 14-day period, from 9 to 22 Nov 2018 in the Sacramento Valley. PM_2.5_ is but one aerosol component of wildfire smoke (WFS)^4^ but is a significant one: because of the particles’ small diameter, they can deposit more deeply in lung tissue, creating more serious damage^5^. PM_2.5_ also contains a mixture of metals, organic carbon, potassium, geologic material, and potentially ammonium nitrate^6^ making it an ideal proxy measure for damage that can be caused by WFS. Because large number of structures (almost 19,000) were destroyed in the Camp Fire, including most of the town of Paradise, the smoke from this fire contained an unusual mixture of components:; Willson et al.^7^ reported high levels of phthalates, and a recent report by the California Air Resources Board^6^ found significantly elevated levels of lead and zinc in the Camp Fire smoke associated, presumably, with the combustion of anthropogenic objects, such as houses, cars, and other objects containing plastics. Recent evidence suggests wildfire smoke (WFS) may also contain infectious microbes^8^.

Along with the greater general concern about wildfires has come a growing interest in the biobehavioral and health consequences of human exposure to wildfire smoke (WFS). Earlier work focused on respiratory consequences of WFS exposure, but more recent studies have found elevated markers of systemic inflammation, such as C-reactive protein (CRP) and interleukin-12 (IL-12), increased risk for all-cause mortality, lower birth weight, and a variety of central nervous system consequences^9–13^. Of particular interest are consequences of exposure to WFS in the prenatal period. Two human^14,15^ and one monkey study^7^ demonstrated that WFS exposure during pregnancy can have reproductive consequences, such as lower fertility, preterm birth, and lowered birth weights, depending on the timing of exposure. No studies exist, however, examining biobehavioral consequences for infants of WFS exposure in utero. A related literature examining prenatal exposure to air pollution (which contains many of the same components as WFS including elevated PM_2.5_), however, has shown links between prenatal exposure and measures of attention deficit hyperactivity disorder (ADHD), reduced left hemisphere white matter, conduct disorder, poor emotion regulation, decreased corpus callosum volume, poor memory and attention, and autism spectrum disorder (ASD)^16–21^. Given the more transitory nature of WFS compared to air pollution, might prenatal exposure produce similar outcomes?

In the present report, we took advantage of an ongoing BioBehavioral Assessment (BBA) program at the California National Primate Research Center to examine the effects of prenatal exposure (specifically, exposure in the first third of pregnancy) to WFS from the Camp Fire. We were interested in three specific questions. First, were the pregnant mothers’ WFS exposure associated with behavioral and physiological consequences to their infants? Subjects were rhesus monkeys that lived outdoors year-round. The period of active smoke exposure was 9–22 November 2018, which was approximately at the peak of the six-month breeding season. Conception dates for these 2018 pregnancies were estimated by subtracting 165 days from the date of birth and ranged from 9 October to 27 December 2018. All animals in the target cohort were classified as “exposed” or “not-exposed” in utero based on whether the conception date occurred, respectively, on/before 22 Nov 2018 (exposed, *n* = 52) versus after that date (non-exposed, *n* = 37). An alternative hypothesis for any group differences that we found, however, could simply be that they were a consequence of conception timing within the breeding season: animals conceived early in a breeding season (which typically runs from September through December every year) are simply different from animals conceived later in a breeding season. Our second question then was, could the differences found be explained simply by conception timing effects? To answer this question, we drew a large historical control cohort (*n* = 2490) from the previous 18 years of the BBA program and classified animals in identical fashion based on estimated conception dates corresponding to the end of the WFS in the target cohort: animals in the control cohort were classified as “early” if their conceptions occurred between 9 October and 22 November, and “late” if their conceptions occurred between 23 November and 27 December, of their respective years. This larger sample size provided good statistical power to detect even small conception timing influences. Finally, the group differences found while investigating the first question raise the third question: given group (exposed vs. non-exposed) differences in response to the first analysis, which of the two groups is actually the adversely affected group? Because oocyte growth and maturation in human and nonhuman primates is a months-long process prior to ovulation and involves epigenetic reprogramming of the oocyte^22^, we cannot rule out that effects of WFS exposure persisted beyond the period of intense smoky air and could affect fetal development in our non-exposed animals. Such latent effects, both reproductive and behavioral, have been found, for example, in a primate model of binge drinking, in which the drinking ended prior to conception^23,24^. Consequently, we used the larger, historical cohort to answer this question. Because no group (early vs. late conception) differences were found in the control cohort in answer to our second question, we combined groups and calculated confidence intervals (CI) for each outcome measure. We separately calculated CI for the exposed and for the non-exposed animals in our target cohort and examined the degree of overlap with the CI of the control group to show the extent to which the two groups in the target cohort were similar or dissimilar to the larger control cohort.

Based on data reviewed above, we expected that exposed animals would show evidence of elevated systemic inflammation, reduced behavioral responsiveness to stress, impaired cognitive function, and altered corticosteroid concentrations compared to non-exposed animals. We expected no differences in any measures based on conception timing (early vs. late) within our control cohort, and expected that the exposed animals in our target cohort would be the group that is most different from the controls. Here we show support for all three expectations.

## Results

### Air quality

**Fig. 1:**
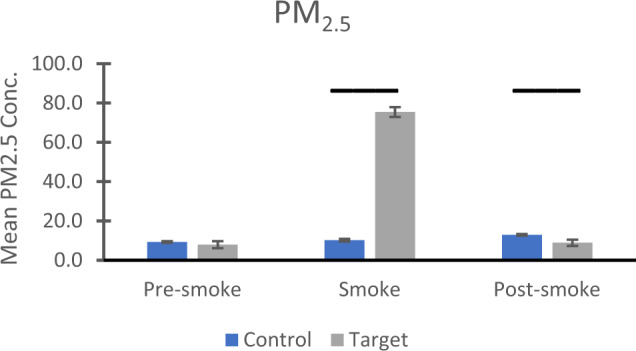
Air quality in Davis/Woodland, California. Mean and standard errors for PM_2.5_ values (in µg/m^3^) for three time periods based on estimated conception dates: pre-smoke (9 October–8 November), smoke (9–22 November), and post-smoke (23 Nov–27 December). Control group refers to animals conceived between 2000-2017 inclusive; target group pertains to animals conceived in 2018. Values shown are from raw data; for statistical analysis, values were log_10_-transformed. Bars show significant, Bonferroni-corrected, follow-up effects from a significant interaction with the transformed data. Higher PM_2.5_ concentrations were seen in the target group during the smoke period (*p* < .001) and in the control group in the post-smoke period (*p* = .009). For the control years (conceptions during 2000-2017), number of observations were 433, 213, and 514 for pre-smoke, smoke, and post-smoke. For the target year (conceptions in 2018), observation numbers were 28, 14, and 35 for the same three conditions, respectively. Source data are presented in a Source Data file.

Daily PM_2.5_ data were downloaded from an EPA website for every day between 9 October and 27 December for every year from 2000 to 2018, and were organized into three time periods based on conception dates determined by events in 2018: pre-smoke (conceptions between 9 October and 8 November), smoke (conceptions 9–22 November), and post-smoke (conceptions between 23 November and 27 December). (Data are available as Supplementary Data File 1) Means and standard errors for the raw data are presented in Fig. 1. After log_10_ transformation, two-way analysis of variance revealed significant effects for period (F(2,1231) = 50.203, *p* < .001, η_p_2 = .075), cohort (F(1,1231) = 33.555, *p* < .001, η_p_2 = .027), and the period by cohort interaction (F(2,1231) = 56.169 *p* < .001, η_p_2 = .084). Follow-up tests for the period main effect revealed that for the entire sample, PM_2.5_ levels were significantly higher in the smoke period compared to the pre-smoke (*p* < .001) or post-smoke periods (*p* < .001). Similarly, the target cohort experienced significantly higher PM_2.5_ compared to the control cohort (cohort main effect). Evaluation of the significant interaction showed no significant differences between the control and target cohorts for the pre-smoke period (*p* = .281), significantly greater PM_2.5_ exposure for the target compared to the control cohort for the smoke period (*p* < .001), and significantly greater PM_2.5_ exposure for the control cohort, compared to the target cohort, for the post-smoke period (*p* = .009).

### Exposed monkeys are different from non-exposed animals

**Fig. 2:**
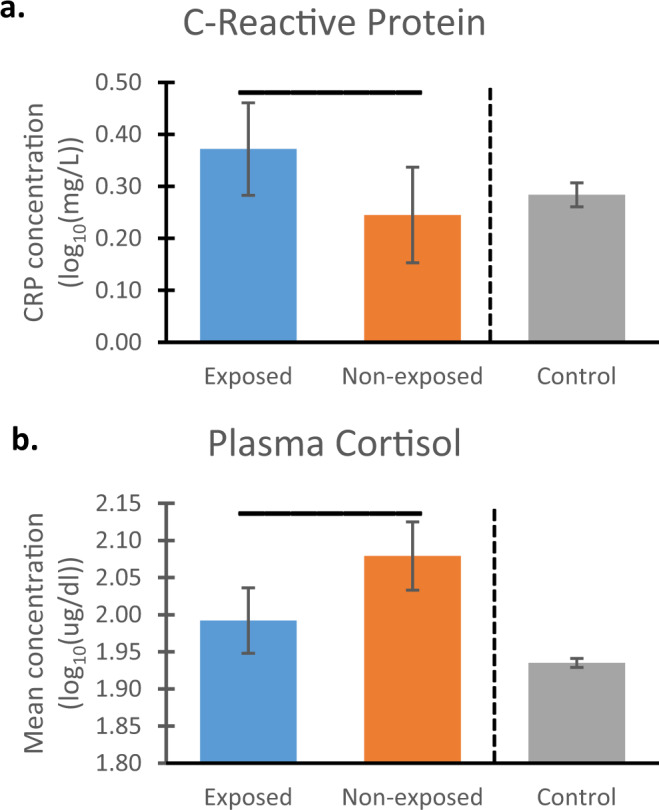
Physiological measures. Statistical comparisons between monkeys exposed vs. non-exposed (blue and orange bars, respectively) to wildfire smoke prenatally show exposed animals had (**a**) elevated mean levels of C-reactive protein (CRP; measured as milligrams/Liter) and **b** reduced mean concentrations of plasma cortisol (measured as micrograms/deciliter), compared to monkeys that were not prenatally exposed. Data were log_10_ transformed; error bars reflect 95% Confidence Intervals (CI), and values are adjusted for the covariate. Overhead bars indicate significant effects: for CRP, *p* = .05 (two-tailed), and for cortisol, *p* = .008. To the right of the dotted lines are the corresponding values for the full control cohort (mean, 95% CI). Comparison of the overlap in CI between the groups in the target cohort with the control cohort revealed that, for CRP, control animals’ CI show complete overlap with those of non-exposed animals from the target cohort and less overlap with those from exposed animals. For cortisol, there is no overlap between the CI for the control cohort and for either group in the target cohort. For CRP, sample sizes are *n* = 40, 31, and 317 for exposed, non-exposed, and control, respectively; For cortisol, the numbers are 40, 31, and 2490, respectively. Source data are presented in a Source Data file.

Exposed and unexposed animals in our target cohort differed significantly in all domains examined with effect size indices suggesting medium effects^25^ (which corresponds to η_p_^2^ = .06). (All data are available as Supplementary Data File 2) Exposed animals had higher levels of CRP in blood (F(1,66) = 3.995, *p* = .050, η_p_2 = .057) as well as a significantly lower cortisol response F(1,66) = 7.443, *p* = .008, η_p_2 = .101) compared to non-exposed animals. (Table 1 shows sample sizes for all analyses.) The left two bars in Fig. 2a, b show log_10_-transformed mean values plus 95% confidence intervals (CI) for CRP and cortisol, respectively.

**Table 1:** Sample sizes for all statistical analyses.

	Target cohort			Control cohort		
	Exposed	Non-exposed	Totals	Early	Late	Totals
Total #	52	37	89	1812	678	2490
CRP	40	31	71	214	103	317
Cortisol	40	31	71	1812	678	2490
Vis Recog Mem	52	37	89	1744	637	2381
Holding Cage	52	37	89	1812	678	2490
Human Intruder	52	37	89	1809	677	2486
Temperament	52	37	89	1802	678	2480

**Fig. 3:**
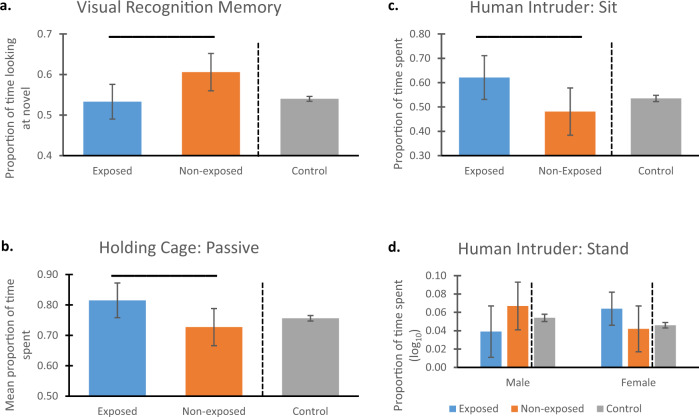
Memory and behavioral data. Statistical comparisons between monkeys exposed vs. non-exposed (blue and orange bars, respectively) to wildfire smoke prenatally show that, compared to non-exposed animals, those exposed to WFS (**a**) had significantly poorer performance on a visual recognition memory task (*p* = .023), **b** showed significantly more passive behavior (*p* = .037), **c** showed significantly more sitting (*p* = .039), and **d** showed a significant interaction of exposure condition by sex for stand, (*p* = .041) (no follow-up comparisons were significant). Overhead bars indicate significant differences. All values shown are means of raw data (except for stand, which was log_10_-transformed) and 95% Confidence Intervals (CI). All values are adjusted for the covariate. Data for **b** were from the Holding Cage observations, and data for **c** and **d** were from the Human Intruder assessment. To the right of the dotted lines are the corresponding values for the full control cohort (mean, 95% CI). Comparison of the overlap in CI between the groups in the target cohort with the control cohort revealed that, for the memory task **a**, controls showed complete overlap with the exposed animals, but did not overlap with non-exposed animals; neither the control nor non-exposed animals’ CIs included the chance response value of 0.5, however. For passive **b**, CI of control animals overlaps completely with those from the non-exposed group; there is less overlap with CI in the exposed group for passive. **c** Controls’ CI overlap completely with those in the non-exposed group, and less completely with the CIs in the exposed group. **d** CI for control animals overlapped completely with both groups from the target cohort for males, but there was less overlap between controls and exposed females. For the exposed, non-exposed, and control groups, respectively, sample sizes were *n* = 52, 37, 2381 for visual recognition memory; *n* = 52, 37, and 2490 for passive; and *n* = 52, 37, and 2486 for sit and stand. Source data are presented in a Source Data file.

Our test of Visual Recognition Memory revealed that exposed animals showed significantly lower performance compared to the non-exposed animals: F(1,84) = 5.355, *p* = .023, η_p_2 = .060). The mean value for the non-exposed animals was .6061, which was significantly different from chance responding of .50 (t(36) = 5.393, *p* < .001). In contrast, the mean for the exposed animals was .5226, which was not significantly different from chance (t(51) = 1.076, *p* = .287). Figure 3a (left two bars) show means and 95% CI for the two groups in the target cohort.

Analysis of behavioral data suggested greater passivity among exposed animals compared to non-exposed animals. In the Holding Cage, exposed animals showed significantly more passive behavior (sit, crouch, and hang) (F(1,84) = 4.479, *p* = .037, η_p_2 = .051; Fig. 3b). Groups did not differ on avoidance or active behaviors, or on coo or bark vocalizations. During the Human Intruder test, exposed animals also showed significantly more sitting (F(1,84) = 4.398, *p* = .039, η_p_2 = .050; Fig. 3c), and less environmental exploration (Mann–Whitney *U* = 759.0, *p* = .023). An interaction of exposure condition by sex was found for stand (which was log_10_-transformed: F(1,84) = 4.308, *p* = .041, η_p_2 = .049; Fig. 3d). While tests of simple effects were not significant, the greatest contrast was for males: exposed animals showed less standing than did non-exposed males. Females showed the opposite pattern, with exposed animals showing more standing than non-exposed females. No group differences were found for active, hang, or bark in the Human Intruder test.

**Fig. 4:**
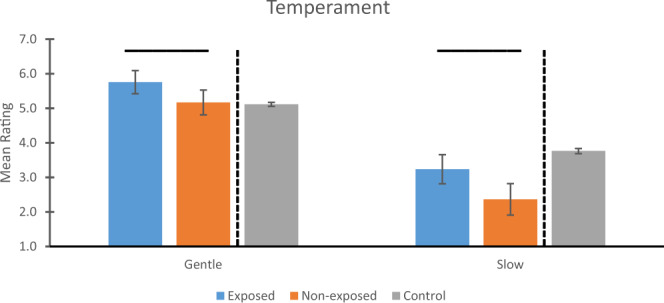
Temperament. Statistical comparisons between monkeys exposed vs. non-exposed (blue and orange bars, respectively) to wildfire smoke prenatally show that, compared to non-exposed animals, those exposed to WFS were rated more Gentle (*p* = .020) and more Slow (*p* = .006) compared to animals not exposed. Overhead bars indicate significant differences. Mean ratings and 95% Confidence Intervals (CI) are shown for Gentle (left panel) and Slow (right panel). All values are adjusted for the covariate. To the right of the dotted lines are the corresponding values for the full control cohort (mean, 95% CI). Comparison of the overlap in CI between the groups in the target cohort with the control cohort revealed that, for Gentle, there was no overlap in CI between controls and exposed animals, but complete overlap for controls and non-exposed animals. For Slow, both exposed and non-exposed animals in the target cohort had CI that were below those of the control cohort’s CI. Sample sizes for both temperament measures were *n* = 52, 37, and 2483 for exposed, non-exposed, and control groups, respectively. Source data are presented in a Source Data file.

Finally, the idea that the exposed animals were more passive was supported by the temperament data (Fig. 4). Exposed animals were rated significantly more Gentle (F(1,84) = 5.644, *p* = .020, η_p_2 = .063) and Slow (F(1,84) = 7.813, *p* = .006, η_p_2 = .085) compared to non-exposed animals.

### Group differences are not due to conception timing

We found multiple group differences between exposed and non-exposed animals in our target cohort, but an alternative hypothesis for such differences is that they are simply a result of conception timing within the breeding season. Our control cohort was divided into early vs. late conception groups based on the same date criteria as in our target cohort, and identical analyses were performed to examine this possibility. No statistically significant group differences were found for any measure, including CRP (*p* = .098) and cortisol concentrations (*p* = .966) (Supplementary Fig. 1A, B).

No significant group differences were found for Visual Recognition Memory (*p* = .609), and mean values for both early and late conception groups were significantly greater than chance responding of .50 (both *p* < .001) (Supplementary Fig. 2A).

Similarly, no significant group differences were found for any behavioral measure: in the Holding Cage, groups did not differ in passive behavior (*p* = .611) (Supplementary Fig. 2B). For the Human Intruder assessment, parallel analyses were also not significant: sit (*p* = .495), environmental exploration (*p* = .264), and stand (interaction: *p* = .499) (Supplementary Fig. 2C, D).

Finally, early versus late conception groups did not differ significantly on the temperament traits of Gentle or Slow (*p* = .643 and *p* = .660, respectively) (Supplementary Fig. 3).

### Exposed animals are largely the affected group

Because there were no significant group differences in our control cohort based on conception timing, we combined the early and late groups and calculated the mean and the 95% confidence intervals (CI) to compare with the exposed and the non-exposed animals from our target cohort. These values appear to the right of the vertical dotted lines in Figs. 2–4. In general, there was less complete overlap in CI between the exposed and control animals than there was between the non-exposed and control animals, suggesting that, in most cases, the exposed animals were the affected group.

For CRP, the CI for control animals completely overlapped with the non-exposed animals’ CI values, but showed less overlap with those from the exposed group (Fig. 2a). Figure 2b indicates no overlap between the CI for the control cohort and either group in the target cohort, with cortisol levels for the controls lower than for those of either the exposed or non-exposed animals.

On the memory task, inspection of the confidence intervals (Fig. 3a) indicates that controls showed complete overlap with the exposed animals, but did not overlap with non-exposed animals. Controls’ performance was significantly above chance levels, however, as was that of the non-exposed animals; performance by the exposed animals, however, was statistically at chance responding.

For the Holding Cage observations, Fig. 3b indicates complete overlap of CI for the controls with the non-exposed animals for passive; the controls showed partial overlap with the exposed animals for passive behavior. For the behaviors from the Human Intruder assessment, Fig. 3c shows complete overlap between controls and non-exposed animals, and only slight overlap in CI between control and exposed animals for sit. Figure 3d shows a similar pattern, with complete overlap between controls and both target groups for males, as well as complete overlap in CI between control and non-exposed females; exposed females showed less overlap in CI with controls.

Finally, for the temperament measures, Fig. 4 indicates that, for Gentle, there was no overlap in CI between controls and exposed animals, and complete overlap between controls and non-exposed animals. In contrast, for Slow, there was no overlap between controls and either group in the target cohort.

## Discussion

These data demonstrate a pervasive impact of exposure to WFS in utero on measures of biobehavioral function in infant rhesus monkeys, and the statistical analysis revealed that most of these effects were of medium size^25^. Compared to non-exposed animals in our target cohort, animals exposed early in pregnancy to smoke from the Camp Fire showed, as expected, elevations in a marker of inflammation, an altered cortisol response to stress, memory deficits, and a more passive behavioral response. Only one group by sex interaction was found, for stand. Our second question concerned whether the effects seen in our target cohort could be due simply to timing effects of conception within the breeding season. Despite the much larger sample size and attendant statistical power, no parallel analysis in the control cohort was statistically significant. Finally, comparison of the CI between the control cohort and the two groups in the target cohort suggested that the impact of WFS appears greatest for those directly exposed to WFS, and not for animals conceived after the smoke had cleared: for most of the measures, there was complete overlap between the CI for the non-exposed and the control animals (that is, the control animals’ CI were completely contained within the CI of the non-exposed animals). This was true for CRP, passive, sit, stand (for both males and females), and Gentle. In contrast, only partial or no overlap in CI was found for exposed animals for CRP, cortisol, passive, sit, stand (for females), Gentle, and Slow. While this comparison suggests that many of the group differences in our target cohort were due to biobehavioral changes among the exposed, and not the non-exposed, infants, we note that, for two measures, cortisol and Slow, neither the exposed nor non-exposed groups overlapped in CI with the historical controls, suggesting that there may have been persisting effects of the WFS on fetuses conceived after the smoke had cleared on 22 November 2018.

Many of our findings are consistent with results reported in the literature for exposure to WFS or air pollution, although the majority of the studies were focused on the consequences of direct (i.e., postnatal) exposure. Our finding of higher concentrations of CRP among exposed animals was expected: studies of postnatal exposure to either air pollution^9^ or WFS^12^ have demonstrated increases in measures of systemic inflammation. Regarding the cortisol results, while our findings showed that non-exposed animals had higher cortisol compared to the exposed animals, comparison of the confidence intervals revealed no overlap between the exposed and the control animals, suggesting that WFS elevated cortisol concentrations in the exposed animals (see below for more discussion of the glucocorticoid results). Many studies have shown that exposure to particulate matter produces elevations in corticosteroid concentrations (e.g.,^26^), and one study showed a positive association between pregnant womens’ exposure to air pollution and cortisol concentrations in cord blood^27^. Poorer performance on our Visual Recognition Memory task among exposed animals is reminiscent of studies of individuals exposed to air pollution, where deficits, particularly in short-term memory, as was measured in the present study, were found^28^. Interestingly, one study^29^ demonstrated memory deficits that were correlated with maternal plasma levels of the pro-inflammatory cytokine, IL-6, and a recent review suggested elevated inflammation from WFS may be a mechanism resulting in Alzheimer’s Disease^13^, all of which suggest that the effects on cognition of exposure to particulate matter may be associated with inflammation. Finally, we are aware of no studies that have examined how WFS exposure might affect behavioral or temperament outcomes, although studies exist focusing on air pollution and behavior, including suggestions of perinatal exposure and elevated risk for autism spectrum disorder^30^. One exception was a field study of wild Bornean orangutans, in which increases in resting (i.e., passive) behavior were found during wildfire conditions, but also persisted into the post-smoke period^31^. Our findings extend this observation into the next generation.

Identification of the mechanisms by which WFS may result in adverse effects on the fetus is beyond the scope of this study, but we suspect involvement of the fetal adrenal. The absence of overlap in the CIs for the exposed animals and the controls suggest elevated cortisol concentrations in the exposed animals, which we attribute to changes in the fetal environment as a result of mothers’ exposure to smoke. This effect likely involves the placenta, which, at this point in rhesus monkey fetal development, provides endocrine support to the fetal adrenals to make androgens for conversion of pregnancy estrogens and to make estrogen-receptor-beta ligands for brain development. We suspect, based on past work^32^, that components of WFS, like chlorinated hydrocarbons, may target placenta invasion and proliferation, fetal adrenal programming, and brain development. In fact, elevated glucocorticoids in the prenatal period have been associated with up-regulation of glucocorticoid-responsive genes in brain^33^ and stress-induced exposure to cortisol early in gestation revealed adverse effects on behavioral and cognitive function^34^ and temperament^35^ in infancy in humans. In short, we propose that the effects of WFS early in development are more likely to be a result of teratogenicity of WFS components than stunting of fetal growth and development.

Animals in the non-exposed group also had high levels of cortisol, in fact significantly higher than for animals in the exposed group, but did not show the behavioral effects that the exposed animals did. We hypothesize that elevated cortisol in this group is associated with lingering effects of the WFS in either the mother’s or the father’s reproductive systems. However, because oogenesis is a months-long process in female rhesus monkeys^22^ we consider it more likely that the elevated cortisol in the non-exposed group may be due to the father’s exposure, insofar as spermatogenesis occurs within a much shorter time frame; one estimate is that the total duration is 36 days^36^. In other work^37^, we have shown that early stress on males, but not females, can lead to elevated cortisol responses of the offspring during the BioBehavioral Assessment. Based on these data, we propose that the father’s effect may not have led to elevated cortisol in the prenatal period per se, but rather to epigenetic alterations in adrenal responsiveness, such as in the stress of participation in the BBA program. The lack of elevated cortisol while in utero could explain why we did not see the behavioral effects that were evident in the exposed group. An alternative mechanism may involve inflammation, inasmuch as we found higher levels of CRP in the exposed than in the non-exposed animals, and other research has shown that elevated inflammation in the prenatal period is associated with neurodevelopmental compromise^38^. We acknowledge, of course, the speculative nature of this discussion and recognize that a better understanding of the mechanisms requires targeted study.

This report shows that WFS exposure early in gestation impacts measures of biobehavioral organization in infant rhesus monkeys and raises a number of additional questions. First, it will require future research to know which of the components of WFS are toxic to biobehavioral development. Willson et al.^7^ and the California Air Resources Board^6^ showed how the chemical composition of the PM_2.5_ particles changed during the course of the Camp Fire, and others have shown, for example, that water-soluble inorganic components of PM_2.5_ such as nitrate have especially strong influences on activation of the hypothalamic-pituitary-adrenal axis^39^. Future studies involving controlled experimental exposure to wood smoke or wood smoke components would be informative. Second, we don’t know from our data whether there are particularly sensitive periods during the prenatal period when exposure might be especially detrimental. In our target cohort, estimated conception dates indicate that the earliest-conceived subjects were 44 days of gestational age, still well within the first third of the 165 day gestation, on the final smoky day. Other studies have shown differential effects of prenatal exposure to ketamine^40^ or to stress^41^ between those exposed in the first versus the second third of pregnancy in rhesus monkeys. Using a controlled exposure paradigm, with known conception dates, as just described, can help determine if and when such sensitive periods may exist. Third, we cannot easily disentangle the effects of pre-conception exposure. Recall that our exposed animals were those conceived on 22 Nov 2018 (the last smoky day) or earlier. The mother of an animal conceived on that exact date would have herself been exposed to 13 previous days of WFS prior to conception. How might this have affected the conceptus? Similarly, we do not know how long any persisting effects of WFS may have occurred in the non-exposed cohort, although the elevated cortisol in this group suggests that there were some lingering effects, as suggested above. These are important questions that can best be answered by controlled, timed exposures with known conception dates. Finally, it is important to know if prenatal WFS exposure might have biobehavioral consequences in later life, as others have shown for reproductive and immune outcomes following post-natal WFS exposure^42^. Moreover, previous work has indicated that poor performance on our memory task in infancy (which characterized the exposed animals in our target cohort) is strongly associated with poor social functioning years later^43^. These results suggest domains to explore in follow-up studies of these prenatally exposed animals.

We recognize several limitations of our study. First and foremost, our study used estimated conception dates, inasmuch as the actual conception dates are impossible to obtain in our large outdoor cages. Prospective studies could make use of time-mating protocols on indoor housed animals to get more precise conception dates. Second, as noted above, WFS is a dynamic amalgam of many different components, whose complexity is not captured fully by PM_2.5_ measures, such as was used here. It remains to be explored how phthalates, nitrates, and other components of WFS contribute to infant development. Third, we acknowledge the heterogeneity of our control cohort. While none of the animals experienced a prenatal exposure to WFS like those in our target cohort, some were likely conceived after smoke had cleared from pre-breeding-season wildfires in the area; consequently, any lingering impact on the reproductive systems of dams and sires could have affected some animals in our control cohort. Similarly, animals in the control cohort are likely to have experienced bad air due to other events, such as smog and layer inversions. Consequently, we acknowledge that the data from our control cohort are noisy. Finally, we recognize that, given the situation resulting from a major event like the Camp Fire, it was virtually impossible for our technicians to be blind to the exposure status of animals from the 2018 conception year. They were, however, blind to our particular hypotheses. As mentioned earlier, future studies involving controlled exposures with a properly drawn control cohort, could mitigate or eliminate many of these concerns.

In conclusion, our data suggest that exposure to WFS in the first third of pregnancy has lasting consequences for biobehavioral development in infant rhesus monkeys. We recognize that the majority of humans are not exposed continuously to WFS as were our monkeys, though near-continuous exposure probably characterizes many throughout the world who have inadequate shelter to escape smoke (and some evidence indicates smoke can penetrate indoors^44^), including growing homeless populations in developed countries. Continuous exposure also likely characterizes the experience of wildlife populations across all taxa that live in wildfire areas. The amount and timing of exposure to WFS, as well as the mechanisms involved in creating the adverse consequences, are empirical issues to be determined.

## Methods

The study complied with all relevant ethical and legal regulations, and all procedures were approved by the Institutional Animal Care and Use Committee of the University of California, Davis.

### Subjects and living arrangements

Our target cohort comprised *n* = 89 (56 females) infant rhesus monkeys (*Macaca mulatta*) born in 2019 at the California National Primate Research Center. Mean age of the animals was 109.1 days (SD = 7.96 days, range = 90–124 days) at the time of testing in the BioBehavioral Assessment (BBA) program (see below). Conception was defined as the date of birth minus 165 days, reflecting the mean gestation at our facility^45^, and ranged from 9 Oct 2018 to 27 Dec 2018. Animals were classified as exposed (*n* = 52, 37 females) to WFS if their conception date was 22 Nov 2018 or earlier, and non-exposed (*n* = 37, 19 females) if conception occurred on 23 Nov 2018 or later.

A control cohort of *n* = 2490 animals (1336 females) was drawn from the larger BBA database based on the following criteria: reared in the same outdoor environments as the target cohort; born between 2001 and 2018; and conceived between 9 October and 27 December of the year prior to birth. Animals were classified as in our target cohort, with those conceived between 9 October and 22 Nov (*n* = 1812, 976 females; the early control group) contrasted with animals conceived from 23 Nov to 27 Dec (*n* = 678, 360 females, the late control group) to match the exposed and non-exposed groups in 2018. Mean age was 109.2 (SD = 9.33, range = 88–133 days) at time of BBA testing.

All animals were born and reared in outdoor field corrals built of pipe and chainlink, and measuring 0.2 ha in area, each containing up to 200 animals of all ages, with a population structure approximating that seen in the wild. Animals were fed twice daily, water was continuously available, fresh produce was given once or twice per week, and each cage contained a variety of climbing and shelter structures.

### Air quality

Mean daily values for PM_2.5_ from 2003 to 2018 were obtained from a dedicated air quality monitoring site on the University of California, Davis campus, operated by the California Air Resources Board (https://www.epa.gov/outdoor-air-quality-data/download-daily-data). The monitoring instrument is a Met One BAM 1020. For 2000–2002 PM_2.5_ data, we relied on data from a site located in Woodland ~19 km from CNPRC, which used Anderson RAAS2.5-300 and R&P Model 2000 units for PM_2.5_ measurements. For the years 2003 and 2004, we had data for *n* = 48 dates from both the Davis and Woodland sites, which correlated strongly (*r* = 0.957, *p* < .001) with no mean difference (t(47) = −1.030, *p* = .308). Consequently, we combined the data from the two locations to assess air quality for our entire control cohort from 2000 to 2017.

### BioBehavioral Assessment (BBA) program

Details of the BBA program have been published^46,47^. Briefly, mothers and infants were net captured from their field corrals, separated from each other, and delivered to the indoor testing room (infants) or to holding cages (mothers) that were outside sensory range of the infants. Three-to-four month old infants, which were always tested in cohorts of 5–8 animals, arrived at 0900 h, and were housed individually in standard-sized holding cages (0.58 m × 0.66 m × 0.81 m, Lab Products, Maywood, NJ). A variety of behavioral assessments were performed throughout the day and early the next day. Infants were returned to their mothers at 1000 h the following day, where they were given an hour to nurse prior to return to their corrals with their mothers. Each infant holding cage contained a stuffed cloth toy duck, a towel, and a novel object that the infants could manipulate. Infants were provided with water ad libitum, orange-flavored drink, fresh fruit, rice cereal, and commercial monkey chow. Monkeys from the target and control cohorts experienced identical procedures at the same times of day. Four sets of assessments were examined in the present report.

Physiological data were obtained from two blood samples obtained on the first day of BBA testing. Sample 1 (1 ml) was drawn at 1100 h, approximately two hours after subjects were separated and relocated – sample 1, therefore is a stress sample. Sample 2 (0.5 ml) was obtained at 1600 h, and reflects animals’ responses to sustained stress. In all cases, blood was drawn into unheparinized syringes from a femoral vein following manual restraint, and was immediately transferred to tubes containing EDTA. 0.5 ml from sample 1 was delivered to CNPRC’s Clinical Laboratory for analysis of C-reactive protein. The remaining 0.5 ml from sample 1, and the 0.5 ml from sample 2 were spun in a refrigerated centrifuge at 4 °C for 10 min at 1277 g. Plasma was removed and frozen at −80° until assay.

Cognitive data were provided by a test of Visual Recognition Memory, administered at 1130 h on Day 1. Each animal was hand-carried to a test cage measuring 0.387 m × 0.413 m × 0.464 m that was positioned 0.686 m from a 0.813 m monitor (Panasonic KV 32540), was given 30 s to habituate, and was presented with seven problems from a pre-recorded video. Each problem included three trials, a familiarization trial and two recognition trials. After a 5 s blank screen, a 20 s familiarization trial began, in which two identical pictures were presented, each measuring 19.7 cm × 22.9 cm, separated by 25.4 cm of white space onscreen. After another 5 s delay, an 8 s recognition trial occurred, in which the now-familiar stimulus was presented simultaneously with a novel stimulus (side determined randomly). Following another 5 s delay, the same two stimuli were presented again for 8 s, with positions reversed. Seven such problems were presented. All stimuli were pictures of unfamiliar juvenile and adult monkeys of both sexes (stimuli are available as supplementary material for Sclafani et al.^43^). A tone of 1000 Hz was presented 250 milliseconds prior to trials in order to orient the animal. A low-light camera (Radio Shack Observation 49–2502 through 2004, then KT&C Corporation KTL CMB5010EX), attached to the display monitor and situated midway between the two projected images, was used to record the subjects’ looking responses. For each problem, the proportion of looking time directed at the novel stimulus was computed: duration of viewing the novel stimulus on the two recognition trials divided by the duration of viewing both the novel and familiar stimuli in the recognition trials. The principal outcome measure was a mean of this proportion across the seven problems. Chance responding was indicated by a mean of 0.50, with lower values suggesting a preference for the familiar stimuli, and higher values indicating preference for the novel stimulus. A variety of studies have shown that normal rhesus monkeys show a preference for the novel stimuli, and monkeys that are impaired tend to show no preference, or prefer the familiar stimuli^48^. Upon completion of testing, the subject was returned to its holding cage, and the test area cleaned and prepared for the next subject.

Behavioral data were recorded from two situations, using a standard ethogram for infant rhesus monkeys (see Table 2 in^47^) and The Observer^49^ software package. Inter- and intra-observer reliability were established at 85% agreement or better on behavior categories. Holding Cage observations comprised five minute focal animal observations on each animal (using a pre-determined random order) on two occasions – beginning at 0915 h (15 min after arrival in the test area) and on Day 2 at 0700 h (three hours prior to return to mother). The Day 1 observations reflect initial responses to the separation and relocation, and the Day 2 responses reflect adaptation to the BBA situation. Analyses focused on the proportion of time spent in passive (sit, crouch, hang from side of cage), avoidant (sleep, lie), and active/inquisitive (locomotion, stand) behavior, and rates (per 60 s) of coo and bark vocalizations. The Human Intruder observations were made at 1400 h on Day 1. The Human Intruder test assesses responsiveness to a standardized challenge, and comprises four one-minute trials (a) technician 1 m in front of the animal’s cage (far position), presenting left profile; (b) technician ~0.3 m (near position) with left profile; (c) far position while making direct eye contact with the animal; (d) near position, direct eye contact. Predominant responses on this brief test included time spent sitting, standing, active, and hanging from the side of the cage, and rates of bark vocalization and environment exploration (which, in this context, is an avoidant behavior). For both the Holding Cage and Human Intruder tests, behavioral data were collected as frequencies and/or durations. However, owing to slight variations in the length of each observation session (eg, for the one-minute trials in the Human Intruder test, one animal may have experienced a condition for 58.6 s and another animal for 61.1 s), durations were converted to proportion of time observed by dividing the duration by the length of the observation for that specific animal. Frequencies were converted to rate per 60-sec.

Temperament data were obtained at the end of the 25-h assessment period by a trained observer that did all of the testing with the animals. Each animal was rated on a list of 16 adjectives (listed in Table 3 in^45^) describing affect quality using a Likert-type scale of 1–7, with 1 reflecting a total absence of the trait and 7 reflecting an extremely large amount of the trait.

### Assays

C-reactive protein was assessed using a high sensitivity assay (Beckman Coulter, OSR6199, Brea, CA) according to manufacturer instructions, utilizing CNPRC’s Clinical Laboratory’s Chemistry Analyzer (Beckman Coulter AU480, Brea, CA). Animals were selected only if they had values less than 10 mg/L, which is considered the normal range for our facility. All animals in the target cohort met this criterion, but since CRP was assayed beginning in 2016, only *n* = 317 animals were available in the control cohort for this measure (six animals were excluded whose CRP values were greater than 10.0).

Cortisol concentrations were assessed via I_125_ radioimmunoassay using validated kits from Siemens Corporation through 2013, after which a quantitative competitive immunoassay that employs direct chemiluminescent technology on the ADVIA Centaur CP platform (Siemens Healthcare Diagnostics, Tarrytown, NY, USA) was used. Both assays measure total cortisol. The assay consists of dimethyl acridinium ester labeled cortisol which competes for binding to a polyclonal rabbit anticortisol antibody bound to a monoclonal mouse anti-rabbit antibody covalently coupled to paramagnetic particles. The immune complex is captured and separated by application of a magnetic force. Addition of acid (hydrogen peroxide and nitric acid) and base (sodium hydroxide) reagents produce a chemically induced light emission measured by luminometer in relative light units (RLUs). The RLUs are inversely proportional to the amount of cortisol in the unknown sample. Samples were diluted 1:10 with ADVIA Centaur Multi-Diluent 3 (Siemens Healthcare Diagnostics, Tarrytown, NY, USA) prior to analysis to obtain accurate results. Cortisol data from the chemiluminescent and radioimmunoassay were harmonized as described^50^ using multiple regression based on *n* = 32 samples assayed on both platforms (*R*^2^ = 0.88).

### Data analysis

Data were analyzed (using SPSS v.26) with analysis of variance (using log_10_ transformations as needed) and nonparametric statistics when transformations could not produce homoscedasticity. We expected biobehavioral differences in our target cohort based on WFS exposure (exposed animals were conceived on 22 Nov 2018 or earlier vs. non-exposed animals, who were conceived later than that). Because there was no a priori reason to assume that animals from the control cohort that were conceived late (i.e., after 22 Nov) were different on any of our measures compared to animals conceived earlier, however, we expected non-significant results for all analyses for this cohort, despite the larger sample size and increased power. Because no statistically significant results were found in the control cohort between early and late conceptions (see Results), we combined those groups in order to visually compare, using confidence intervals, control data with the two groups in our target cohort. This comparison enabled us to identify which group in our target cohort – exposed or non-exposed – was most similar or different to data from the control animals.

Sample sizes for all analyses are shown in Table 1. For the target cohort, all analyses involved the full sample of *n* = 89 animals except for the physiological measures (CRP, cortisol), for which blood samples were obtained for *n* = 71 of the 89 animals. Among controls, many analyses had slightly fewer animals than in the full cohort, due to missing data from equipment failure or animals that became ill and had their assessments cut short. The principal exception, as noted above, was for CRP, collection of which began with the animals tested in 2016 (who were conceived in 2015).

Independent variables were exposure condition (for the target cohort: exposed vs non-exposed) or conception timing (for the control cohort: early vs. late), and sex. Specific Pathogen Free (SPF) status (monkeys that have been bred to be free of four viral pathogens^51^) was a covariate in all analyses, as previous work in our laboratory has shown that some of our measures are affected by SPF status. Initial analyses included repeated measures as additional independent variables (two days for Holding Cage; four conditions for Human Intruder; two samples for cortisol). No interactions between the repeated measures and exposure condition were found in any analyses; consequently, mean values were computed across the repeated measure variables, which also served to improve homoscedasticity. Effect sizes are indicated by partial eta-squared (η_p_2); for context, Cohen^25^ considers values of .01, .06, and .14 to represent small, medium, and large effect sizes, respectively. For all analyses, results are presented only for exposure condition (target cohort) or conception timing (control cohort), and for interactions of these variables with sex. Figures show means and 95% confidence intervals for exposed vs. non-exposed groups in the target cohort, and for the full control cohort for comparison. All tests were two-tailed. (We recognize that some readers might prefer three-way ANCOVA, with cohort as a third factor, rather than our preferred separate two-way ANCOVAs for the exposed vs. non-exposed, and the early vs. late, contrasts. We note that such an analysis does indeed replicate our findings; we believe our approach provides greater clarity, however. All data are included as Supplementary Material for the reader’s further examination of the data).

### Reporting summary

Further information on research design is available in the Nature Research Reporting Summary linked to this article.

## Supplementary information

Supplementary Information

Reporting Summary

Description of Additional Supplementary Files

Dataset 1

Dataset 2

## Data Availability

All data supporting the findings of this study, as well as the SPSS code used for the analyses, are available as Supplementary Data Files 1 (PM_2.5_ data) and 2 (biobehavioral data) accompanying this article. Source data for all figures (in text and Supplementary Material) are also included as a spreadsheet file. Source data are provided with this paper.

## Source data

Source Data
