# Supplementary material for: Adverse biobehavioral effects in infants resulting from pregnant rhesus macaques’ exposure to wildfire smoke
Source: Nat Commun. 2022 Apr 1;13:1774. doi: 10.1038/s41467-022-29436-9 (PMC8975955; doi:10.1038/s41467-022-29436-9)
Supplement: Supplementary file 1 — Supplementary Information [file 41467_2022_29436_MOESM1_ESM.pdf]

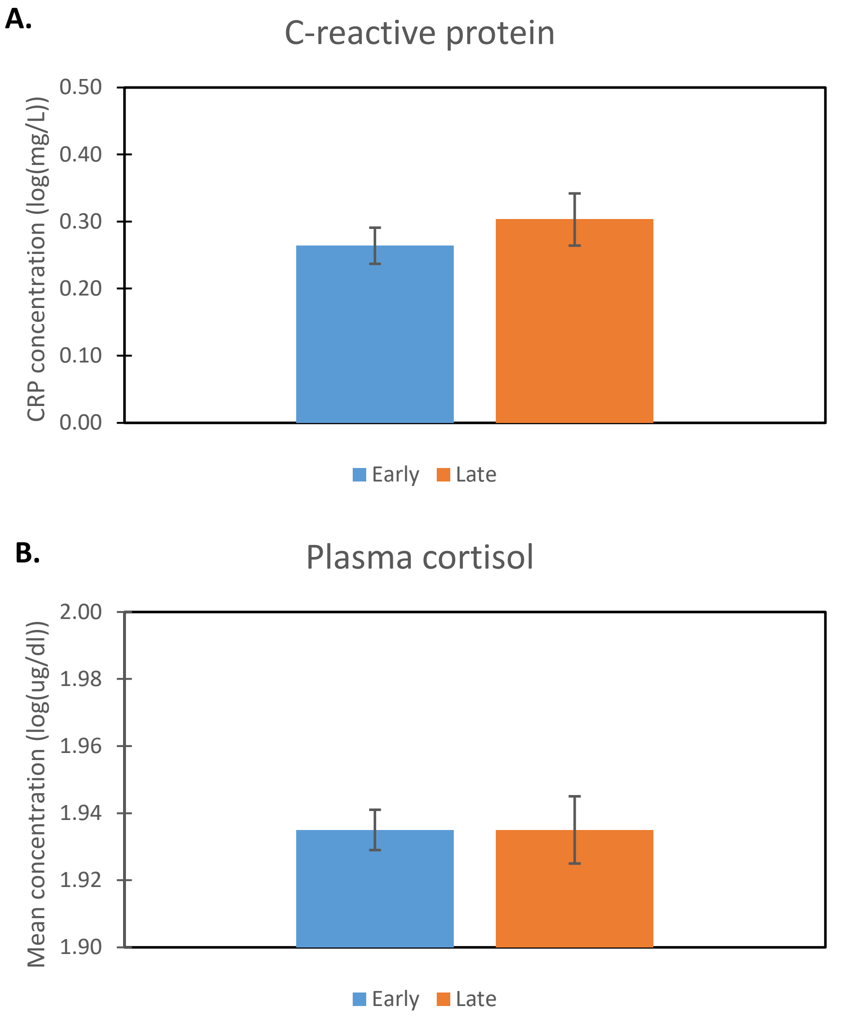

Supplementary Figure 1: Physiological measures. Statistical comparisons between control monkeys conceived early vs. late in the breeding season (blue and orange bars, respectively) showed no significant differences for A) C-reactive protein (p=.098) or B) cortisol (p=.966). Values shown are means, data were log10 transformed, error bars reflect 95% Confidence Intervals, and values were adjusted for the covariate. CRP is measured as milligrams/Liter, cortisol as micrograms/deciliter. Sample sizes for the early and late groups were n=214 and 103 for CRP, and n=1812 and 678 for cortisol.

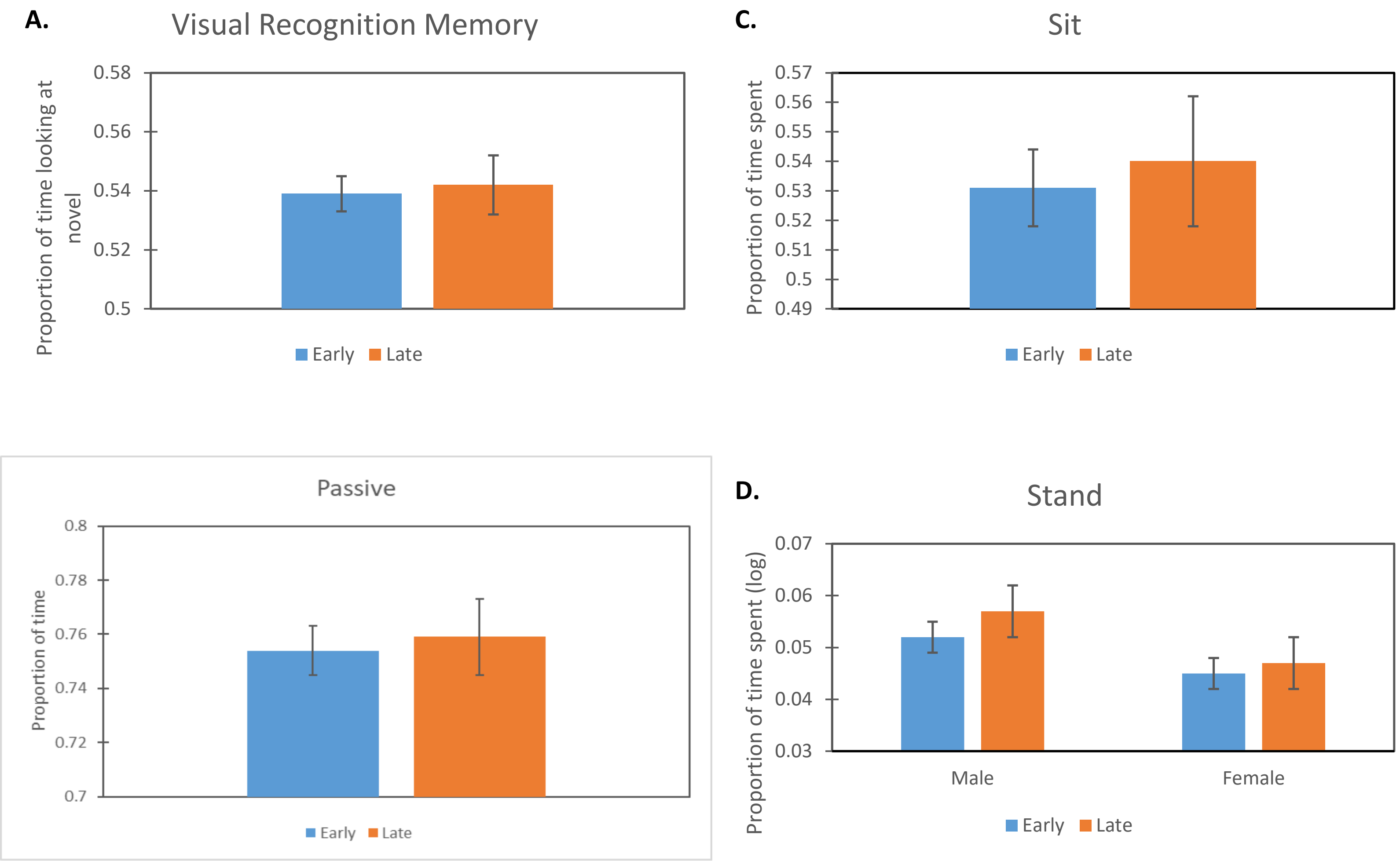

Supplementary Figure 2: Memory and behavioral data. Statistical comparisons between control monkeys conceived early vs. late in the breeding season (blue and orange bars, respectively) showed no significant mean differences for A) performance on the visual recognition memory task (p=.609), B) passive behavior (p=.611) during the Holding Cage observations, C) sit (p=.495) in the Human Intruder assessment, and D) the interaction of exposure condition by sex for stand, (p=.499) All values shown are means of raw data (except for stand, which was $\log_{10}$ transformed) and 95% Confidence Intervals, and values were adjusted for the covariate. Sample sizes for the early and late groups were n=1744 and 637 for visual recognition memory; n=1812 and 678 for passive; and n=1809 and 677 for sit and stand.

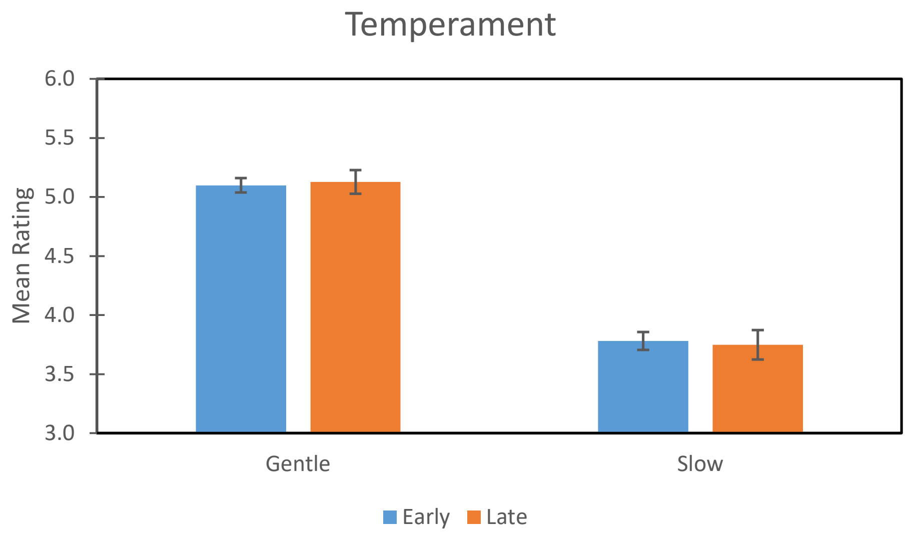

Supplementary Figure 3: Temperament. Statistical comparisons between control monkeys conceived early vs. late in the breeding season (blue and orange bars, respectively) showed no significant mean differences for ratings of gentle (p=.643) or slow (p=.660). Mean ratings and 95% Confidence Intervals are shown for gentle (left pair of bars) and slow (right pair of bars); values were adjusted for the covariate. Sample sizes for the early and late groups were n=1802 and 678 for both gentle and slow.
