# Supplementary material for: Adverse biobehavioral effects in infants resulting from pregnant rhesus macaques’ exposure to wildfire smoke
Source: Nat Commun. 2022 Apr 1;13:1774. doi: 10.1038/s41467-022-29436-9 (PMC8975955; doi:10.1038/s41467-022-29436-9)
Supplement: Supplementary file 2 — Reporting Summary [file 41467_2022_29436_MOESM2_ESM.pdf]

nature portfolio

Corresponding author(s): John P. Capitanio

Last updated by author(s): Jan 28, 2022

Reporting Summary

Nature Portfolio wishes to improve the reproducibility of the work that we publish. This form provides structure for consistency and transparency in reporting. For further information on Nature Portfolio policies, see our Editorial Policies and the Editorial Policy Checklist.

Statistics

For all statistical analyses, confirm that the following items are present in the figure legend, table legend, main text, or Methods section.

n/a	Confirmed	
☐	☒	The exact sample size (n) for each experimental group/condition, given as a discrete number and unit of measurement
☐	☒	A statement on whether measurements were taken from distinct samples or whether the same sample was measured repeatedly
☐	☒	The statistical test(s) used AND whether they are one- or two-sided<br>*Only common tests should be described solely by name; describe more complex techniques in the Methods section.*
☐	☒	A description of all covariates tested
☐	☒	A description of any assumptions or corrections, such as tests of normality and adjustment for multiple comparisons
☐	☒	A full description of the statistical parameters including central tendency (e.g. means) or other basic estimates (e.g. regression coefficient) AND variation (e.g. standard deviation) or associated estimates of uncertainty (e.g. confidence intervals)
☐	☒	For null hypothesis testing, the test statistic (e.g. F, t, r) with confidence intervals, effect sizes, degrees of freedom and P value noted<br>*Give P values as exact values whenever suitable.*
☒	☐	For Bayesian analysis, information on the choice of priors and Markov chain Monte Carlo settings
☒	☐	For hierarchical and complex designs, identification of the appropriate level for tests and full reporting of outcomes
☐	☒	Estimates of effect sizes (e.g. Cohen's d, Pearson's r), indicating how they were calculated

Our web collection on statistics for biologists contains articles on many of the points above.

Software and code

Policy information about availability of computer code

Data collection	Across the 18 years of behavioral data collection reported in this paper, we used The Observer software package, versions 3.0 and 5.0.
Data analysis	SPSS, version 26 was used for all data analyses.

For manuscripts utilizing custom algorithms or software that are central to the research but not yet described in published literature, software must be made available to editors and reviewers. We strongly encourage code deposition in a community repository (e.g. GitHub). See the Nature Portfolio guidelines for submitting code & software for further information.

Data

Policy information about availability of data

All manuscripts must include a data availability statement. This statement should provide the following information, where applicable:

- Accession codes, unique identifiers, or web links for publicly available datasets
- A description of any restrictions on data availability
- For clinical datasets or third party data, please ensure that the statement adheres to our policy

Data generated and analysed for this study are included as supplementary data files.

Field-specific reporting

Please select the one below that is the best fit for your research. If you are not sure, read the appropriate sections before making your selection.

☒ Life sciences ☐ Behavioural & social sciences ☐ Ecological, evolutionary & environmental sciences

For a reference copy of the document with all sections, see nature.com/documents/nr-reporting-summary-flat.pdf

Life sciences study design

All studies must disclose on these points even when the disclosure is negative.

Sample size	For the target cohort, all animals that could be tested were included. For the control cohort, all animals that met the same criteria as animals in the target sample (living conditions, conception dates, participation in the BBA program) were included.
Data exclusions	For the target sample (n=89) all animals' data are included with the exception of CRP and cortisol, because only n=71 of the 89 animals had blood sampled. Among controls, many analyses had slightly fewer animals than in the full cohort (n=2490), due to missing data from equipment failure or animals that became ill and had their assessments cut short. The principal exception to this in the control cohort was for CRP, collection of which began with the animals tested in 2016 (who were conceived in 2015).
Replication	The experiment was performed once and was not replicated, inasmuch as the study design took advantage of a naturally-occurring event, namely the Camp Fire wildfire in northern California in November, 2018.
Randomization	Because we are looking at biobehavioral responses to a naturally-occurring event, exposure to wildfire smoke (WFS), randomization was not possible (eg, we could not randomly assign animals to a WFS year or a non-WFS year). However, during the BBA program each year, animals are selected randomly for testing from our field corrals, inasmuch as more animals are born into the colony each year than we can assess.
Blinding	Again, because we are looking at biobehavioral responses to a naturally-occurring event, wildfire smoke (WFS) exposure, the technician who collected the behavioral data could not be completely blinded to which infants were exposed as fetuses to WFS. However, many cohorts that were tested in 2019 (conceived in 2018) contained both exposed and non-exposed animals, and it would have been difficult to keep track of which animals were exposed or not. Also, the technician was not aware of our hypotheses about WFS. Similarly, the technicians who coded the behavior videos and performed the assays were completely blind to the exposure conditions of the animals.

Reporting for specific materials, systems and methods

We require information from authors about some types of materials, experimental systems and methods used in many studies. Here, indicate whether each material, system or method listed is relevant to your study. If you are not sure if a list item applies to your research, read the appropriate section before selecting a response.

Materials & experimental systems

n/a	Involved in the study	
☒	☐	Antibodies
☒	☐	Eukaryotic cell lines
☒	☐	Palaeontology and archaeology
☐	☒	Animals and other organisms
☒	☐	Human research participants
☒	☐	Clinical data
☒	☐	Dual use research of concern

Methods

n/a	Involved in the study	
☒	☐	ChIP-seq
☒	☐	Flow cytometry
☒	☐	MRI-based neuroimaging

Animals and other organisms

Policy information about studies involving animals; ARRIVE guidelines recommended for reporting animal research

Laboratory animals	As indicated in the manuscript, we studied rhesus monkeys (Macaca mulatta), and all animals were between the ages of 88-133 days, We did not exclude either sex; for the target cohort (n=89), 56 animals were females. For the control cohort (n=2490), 1336 were females.
Wild animals	The study did not involve wild animals.
Field-collected samples	THe study did not involve samples collected from the field.
Ethics oversight	The study complied with all relevant ethical and legal regulations, and all procedures were approved by the Institutional Animal Care and Use Committee of the University of California, Davis.

Note that full information on the approval of the study protocol must also be provided in the manuscript.
