# Supplementary material for: Adverse biobehavioral effects in infants resulting from pregnant rhesus macaques’ exposure to wildfire smoke
Source: Nat Commun. 2022 Apr 1;13:1774. doi: 10.1038/s41467-022-29436-9 (PMC8975955; doi:10.1038/s41467-022-29436-9)
Supplement: Supplementary file 3 — Description of Additional Supplementary Files [file 41467_2022_29436_MOESM3_ESM.pdf]

Title: Supplementary Data 1
Description: Contains the raw PM2.5 data for the Davis/Woodland, California area for all years from 2000-2018. Also contains notes on the variables, and the SPSS code used to analyze the data.

Title: Supplementary Data 2
Description: Contains the physiological and behavioral data from the BioBehavioral Assessment that was done on all animals, from both the target cohort and the control cohort. Also contains notes on the variables, and the SPSS code used to analyze the data.
